# Traditional, complementary, and integrative medicine in the management of ischemic stroke: a narrative review

**DOI:** 10.3389/fphar.2025.1561688

**Published:** 2025-05-30

**Authors:** S. Sowmiya, Rukaiah Fatma Begum, L. S. Dhivya, Praveen Rajendran, N. Harikrishnan, Ankul Singh S

**Affiliations:** ^1^ Department of Pharmacology, Dr M.G.R. Educational and Research Institute, Chennai, Tamil Nadu, India; ^2^ Institute of Pharmaceutical Research, GLA University, Mathura, Uttar Pradesh, India; ^3^ Department of Pharmaceutical Chemistry, Dr M.G.R. Educational and Research Institute, Chennai, Tamil Nadu, India; ^4^ Department of pharmaceutical analysis, Dr M.G.R. Educational and Research Institute, Chennai, Tamil Nadu, India

**Keywords:** ischemic stroke, traditional, complementary, and integrative medicine, neuroinflammation, oxidative stress, complementary alternative medicine

## Abstract

Ischemic stroke remains a leading cause of mortality and long-term disability worldwide, despite advancements in acute intervention and rehabilitation strategies. Traditional, Complementary, and Integrative Medicine (TCIM) systems; including herbal medicine, acupuncture, and mind-body interventions are increasingly being explored as adjunct therapies in stroke management. This narrative review evaluates the current evidence supporting TCIM approaches for ischemic stroke, highlighting their potential neuroprotective, anti-inflammatory, antioxidant, and cerebrovascular effects. Particular emphasis is placed on well-studied botanical interventions such as *Salvia miltiorrhiza* Bunge [Lamiaceae; Salviae miltiorrhizae radix et rhizoma], *Ginkgo biloba* L. [Ginkgoaceae; Ginkgo folium], and *Panax ginseng* C.A. Mey. [Araliaceae; Ginseng radix]. The review discusses the mechanisms of action, clinical trial outcomes, and integration challenges, while underscoring the need for standardization, quality control, and rigorous scientific validation. This work aims to support informed decisions regarding the integration of evidence-based TCIM practices into conventional stroke care protocols.

## 1 Introduction

Ischemic stroke is a sudden neurological impairment triggered by an interruption in the blood supply to a section of the brain. At the molecular level, cerebral blood perfusion causes an acute loss of oxygen and glucose, which lowers the generation of adenosine triphosphate (ATP), causes lactic acidosis, and disrupts cellular homeostasis (Haupt et al., 2023). Ischemic stroke includes an imbalance of ions, abnormal activation of immune cells, and neuroinflammation, which can lead to neuron death (Zhu et al., 2022). It accounts for the second leading cause of death worldwide, accounting for 5.9 million deaths and 102 million disability-adjusted life years lost (Qin et al., 2022a). Several risk factors have been associated with the development of stroke, including diabetes mellitus, smoking, hyperlipidemia, and hypertension (McFarlane et al., 2005). The underlying causes of ischemic stroke primarily involve *in situ* small vessel disease, artery-to-artery embolism, and cardioembolic events (Chen et al., 2019). Clinically, ischemic stroke often presents with sudden-onset symptoms such as non-orthostatic vertigo, slurred speech, diplopia, sensory disturbances (numbness), and unilateral motor weakness or paralysis (Qin et al., 2022b). Interrupting the blood supply causes several pathophysiological alterations that lead to irreversible neural disruption, such as the production of oxygen free radicals and cerebral edema, reactive oxygen species, neuroinflammation, blood-brain barrier (BBB) destruction, and local inflammatory cell infiltration (Wang et al., 2021).

Herbal medicines are being explored as potential neuroprotective agents for ischemic stroke, based on their long history of use in traditional medicine. Plants naturally produce chemical compounds to protect against diseases, predators, and environmental stress. Many of these phytochemicals have anti-inflammatory, antioxidant, and anti-apoptotic properties, which may help in understanding and managing the underlying causes of ischemic stroke (Ranjan, 2023). Acute ischemic stroke can currently be effectively treated with thrombectomy and thrombolysis. However, only a small percentage of patients can benefit from these techniques due to time frame restrictions (Tong et al., 2021; Hurd et al., 2021). Neuroprotective drugs have not worked well in clinical trials because most treatment targets are discovered in animal studies, not in humans. Therefore, there is an urgent need for effective treatments that can be widely used for ischemic stroke (Zhu et al., 2021). Traditional Chinese, Malay, and Indian (Ayurvedic/Siddha/Unani) medicines, as well as homoeopathic remedies, are part of alternative medicine. Acupuncture, homoeopathy, Chinese medicine, Ayurveda, herbal therapies, mind-body practices, and physiotherapy are all included in Complementary alternative medicine (CAM) for stroke (Rajahthurai et al., 2022).

Integrative medicine was established in the 1980s. Using the complementary advantages of macro and micro, global and local, structure and function, traditional and modern, disease differentiation, and syndrome differentiation in Western medicine and Traditional Chisnese Medicine, integrative medicine pioneers have been developing new theories of medicine and pharmacology based on the tenet of “system learning, comprehensively improving mastering, and sorting” (Wang and Xiong, 2012). As integrative medicine continues to evolve, a growing number of stroke centres in China are incorporating TCM to address the increasing demands of the patient population. More standardised and holistic stroke treatment protocols are being developed nationwide. This comprehensive strategy, which combines conventional and traditional therapies, is referred to as integrative medicine rehabilitation (Saceleanu et al., 2023).

An imperative feature of TCM is acupuncture, which involves putting tiny needles into the skin or deep tissues of particular body areas (acupoints). In addition to being safe, effective, cost-effective, and easy to use, acupuncture can be administered by hand, electric stimulation, or warmth. Acupuncture has been shown in numerous clinical and experimental studies to ameliorate neurological impairments caused by ischemic stroke, particularly those related to stroke outcomes. To produce stronger proof, researchers should keep refining the design of clinical trials, expanding the sample size, standardising and quantifying acupuncture procedures, and utilising interdisciplinary approaches (Qin et al., 2022a). The aim of this review is to critically evaluate the current scientific evidence supporting the use of TCIM approaches in ischemic stroke, identify key pharmacological mechanisms and therapeutic targets, highlight limitations in existing studies, and propose directions for future research. This review also assesses the feasibility of integrating evidence-based TCIM practices into conventional stroke management frameworks.

## 2 Literature search strategy

A comprehensive literature search was conducted using four major databases: PubMed/MEDLINE, Scopus, Web of Science, and Google Scholar (for supplementary and grey literature). The search was limited to articles published between January 2000 and January 2025 to capture recent and relevant advances in the field of traditional, complementary, and integrative medicine (TCIM) for ischemic stroke. We used a combination of Medical Subject Headings (MeSH) and free-text terms with Boolean operators such as (“Ischemic Stroke” OR “Cerebral Infarction” OR “Brain Ischemia”) AND (“Traditional Medicine” OR “Complementary Medicine” OR “Integrative Medicine” OR “Ayurveda” OR “Herbal Medicine” OR “Chinese Medicine” OR “Acupuncture” OR “Yoga”) AND (“Treatment” OR “Therapy” OR “Intervention” OR “Management” OR “Neuroprotection”). Only peer-reviewed English-language articles were included, covering *in vitro*, *in vivo*, or human studies that evaluated TCIM interventions specifically for ischemic stroke and discussed mechanisms of action, pharmacological effects, or clinical outcomes. We excluded non-English publications, case reports, editorials, conference abstracts, non-peer-reviewed sources, studies unrelated to ischemic stroke, and those focused on hemorrhagic stroke unless ischemic models were explicitly addressed. The screening process involved an initial review of titles and abstracts by two independent reviewers, followed by full-text screening for eligible studies. Any disagreements during selection were resolved through discussion or consultation with a third reviewer.

## 3 Pathophysiology of ischemia stroke

**FIGURE 1:**
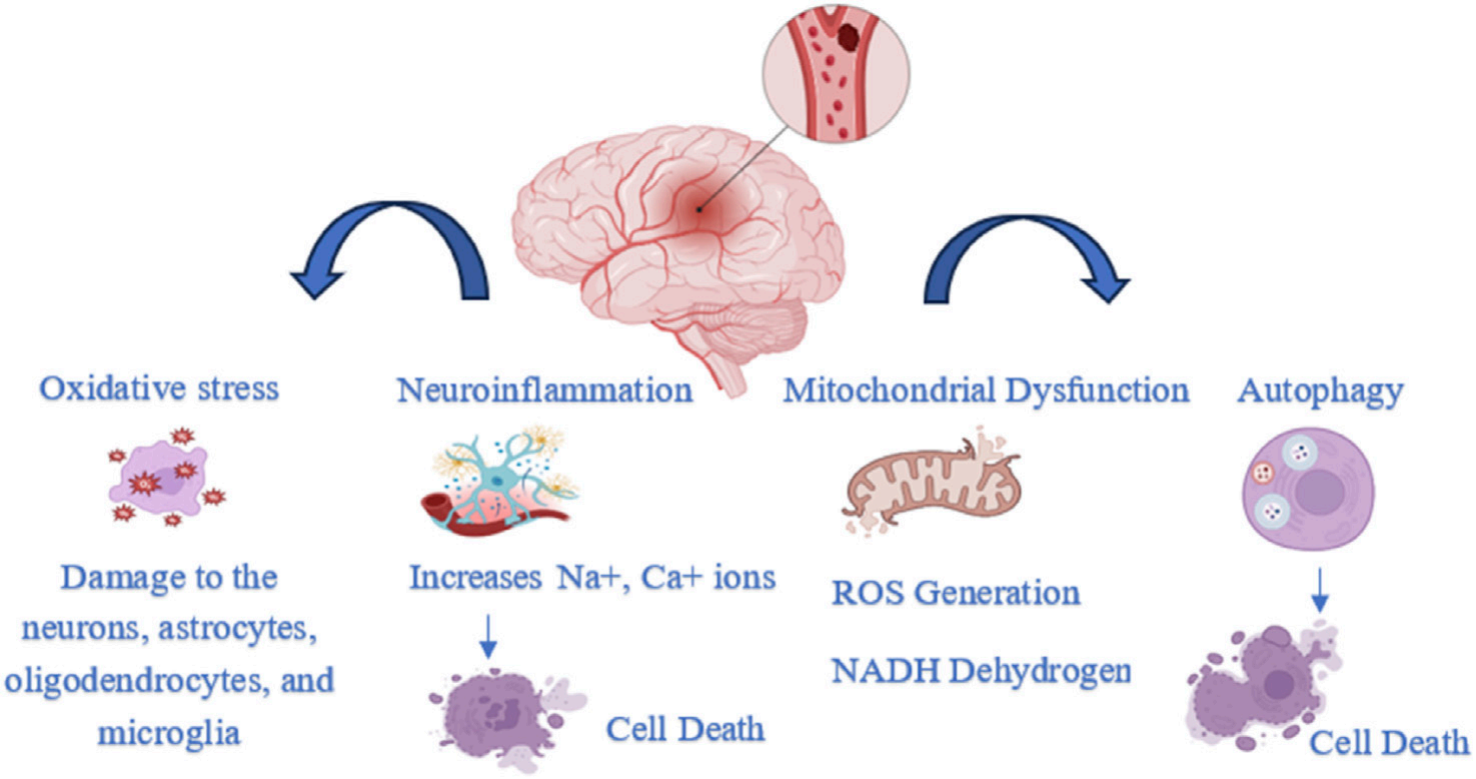
Pathophysiology of ischemia Stroke.

Ischemic occlusions account for approximately 85% of stroke-related deaths, while the remaining cases are due to intracerebral hemorrhage. Ischemic strokes are primarily caused by embolism or brain thrombosis. Beyond the initial vascular occlusion, several secondary pathophysiological mechanisms contribute to the progression of stroke injury. These include inflammation, energy failure, loss of cellular homeostasis, acidosis, elevated intracellular calcium levels, excitotoxicity, cytokine- and free radical-mediated cytotoxicity, complement system activation, disruption of the blood-brain barrier, activation of glial cells, oxidative stress, and leukocyte infiltration (Figure 1) (Kuriakose and Xiao, 2020).

### 3.1 Oxidative stress

Oxidative stress occurs when the production of reactive oxygen species (ROS) exceeds the body’s ability to neutralize and eliminate them (Juan et al., 2021). The brain contains low levels of antioxidant enzymes and cytochrome c oxidase, making it prone to superoxide generation during ATP production. Its high membrane surface-to-volume ratio and lipid-rich plasmalemma increase vulnerability to oxidative damage. Neurotransmitter metabolism leads to calcium overload and ROS formation, while iron released from damaged tissue further promotes free radical production (Jurcau and Ardelean, 2022). ROS are primarily generated as byproducts of mitochondrial electron transport and metal-catalyzed oxidation. Both internal and external factors contribute to their production. Elevated ROS levels are strongly associated with risk factors for ischemic stroke, especially in older adults. (Koutsaliaris et al., 2021). When blood flow is restored to the brain, the return of oxygen can trigger the “oxygen paradox,” leading to a surge in reactive oxygen species that further damages neurons, oligodendrocytes, microglia, and astrocytes (Granger and Kvietys, 2015). Ischemia-reperfusion injury (IRI) causes oxidative stress that leads to endothelial damage, inflammation, and disruption of the blood-brain barrier. This triggers microglial activation, lipid peroxidation, and cell death through processes like ferroptosis, pyroptosis, necroptosis, autophagy, and apoptosis. Long-term damage results from glial scar formation, poor axonal regrowth, ongoing inflammation, and reduced new blood vessel formation and remyelination (Briones-Valdivieso et al., 2024).

Reactive nitrogen species (RNS) are highly reactive molecules produced during nitrogen metabolism. They play key roles in both normal and disease processes, like regulating blood vessel function. Nitrite (NO_2_^−^), a compound made of nitrogen and oxygen, can form salts such as sodium nitrite (NaNO2) or potassium nitrite (KNO2). These nitrite salts, along with SNO-Hb, serve as reservoirs for nitric oxide (NO), which can be released to promote vasodilation and increase blood flow to distant tissues (Salvagno et al., 2024). During ischemia-reperfusion damage in the brain, an increase in both nitric oxide (NO) and superoxide (O2) leads to the rapid formation of peroxynitrite (ONOO) through their direct reaction. Peroxynitrite is more toxic than its precursors. It can nitrify protein tyrosine, forming 3-nitrotyrosine (3-NT), which is believed to carry the signature of ONOO damage (Chen et al., 2018). The generation of reactive compounds like NO_2_^+^, NO2, and OH leads to a series of redox reactions, contributing to secondary nitroxidative stress. Compared to eNOS and nNOS, iNOS produces much higher levels of NO•. This makes iNOS, often called the ‘pathological’ type of NO• synthase, capable of generating ONOO^−^ and highly reactive hydroxyl radicals. NO• plays a critical role in the brain, affecting the neurovascular unit. NO signaling, mediated by constitutive NOS isoforms (nNOS and eNOS), is essential for regulating blood vessel dilation, neuronal excitability, and glial cell function (Wierońska et al., 2021).

### 3.2 Neuroinflammation

Inflammation is another critical factor in ischemic stroke. It worsens nerve tissue damage and cell death through an inflammatory cascade triggered by cerebral ischemia and subsequent reperfusion. This cascade involves oxidative stress, excitotoxicity, inflammatory cell infiltration, and the production of harmful inflammatory mediators (Mo et al., 2020). Consequently, the buildup of Na^+^ and Ca^2+^ ions leads to cell death, membrane damage, and organelle dysfunction. Impaired ATP production also reduces glutamate uptake, causing its excessive accumulation outside cells, which contributes to neuronal death in the ischemic penumbra. The overactivation of glutamate receptors further amplifies excitotoxicity and calcium overload, ultimately leading to mitochondrial failure and cell death (Pawluk et al., 2020). During ischemia, glial cells (microglia, astrocytes), blood cells (like leukocytes), and endothelial cells release various inflammatory mediators, including pro-inflammatory enzymes, cytokines, and chemokines. Alongside these cellular responses, genetic factors that regulate inflammation also significantly contribute to the progression of stroke-related inflammatory processes (Pawluk et al., 2020). During a stroke, astrocytes are rapidly activated by damage-associated molecular pattern molecules (DAMPs) released from injured neurons and glial cells. This activation contributes to blood–brain barrier (BBB) disruption and the recruitment of peripheral leukocytes, driven by proinflammatory cytokines, chemokines, and matrix metalloproteinases such as MMP-9 secreted by reactive astrocytes. These events collectively lead to secondary brain tissue damage (Xu et al., 2020). Following a haemorrhagic stroke, two types of damage occur: primary and secondary. The immune response is believed to play a major role in the secondary phase. Several immunological and inflammatory mechanisms contribute to this stage. For instance, thrombin and blood clots can activate the protease and complement systems, leading to receptor activation and cell lysis. Inflammation during secondary injury can also disrupt the BBB through matrix metalloproteinases (MMPs) and cytokines, increasing capillary permeability and worsening brain edema (Zhao et al., 2022).

### 3.3 Mitochondrial dysfunction

Intracellular organelles like mitochondria have a double membrane and play a crucial role in energy production, cell cycle regulation, and apoptosis. Under low oxygen conditions, the mitochondrial respiratory chain is disrupted, halting ATP synthesis. Electrons accumulate at complexes I and III because they enter complex I more rapidly than they move through complex IV. This imbalance slows the electron transport chain and proton pumping across the inner mitochondrial membrane, ultimately reducing the mitochondrial membrane potential (Jurcau and Ardelean, 2021). Mitochondrial complex I (NADH dehydrogenase), the first component of the mitochondrial respiratory chain (MRC), is strongly implicated in many neurodegenerative diseases due to its role in excessive ROS production and membrane polarization regulation. While complex II (succinate dehydrogenase) also contributes to ROS generation and apoptotic cell death, its involvement in ischemia-induced MRC dysfunction is comparatively limited. Following ischemic injury, both intrinsic and extrinsic apoptotic pathways are activated, involving mitochondrial membrane potential loss, MRC alterations, cytochrome c release, disrupted redox balance, and impaired antioxidant defenses (Reverse Electron Transport at Mitochondrial, 2024).

### 3.4 Autophagy

Controlled cell death is vital for maintaining host defense and tissue homeostasis. PANoptosis, a newly identified form of regulated cell death, integrates features of pyroptosis, apoptosis, and necroptosis. A key link between these pathways is the Caspase family, which plays roles in both pyroptosis and apoptosis, indicating a shared evolutionary origin. Caspases are broadly classified into inflammatory caspases, such as caspase-1, -4, -5, and -11, and apoptotic caspases, such as caspase-3, -6, -7, -8, -9, and -10, reflecting their distinct but interconnected functions (Tian et al., 2025). Pyroptosis is a form of programmed inflammatory cell death driven by caspase-1 activation, characterized by cell swelling and membrane rupture. This process leads to the release of pro-inflammatory cytokines like interleukin-1β and interleukin-18. A key mediator of pyroptosis is the Gasdermin D (GSDMD) protein, which forms pores in the cell membrane, triggering inflammation and cell death. These mechanisms are closely associated with the progression of inflammation-related conditions, including stroke, neurodegenerative diseases, and brain injury (Sun and Zhu, 2023). Apoptosis, triggered through both intrinsic and extrinsic pathways, contributes significantly to neuronal death following cerebral ischemia/reperfusion (I/R). In parallel, necroptosis, a regulated form of necrotic cell death, is primarily controlled by RIPK1, RIPK3, and MLKL. Emerging evidence suggests that necroptosis plays a crucial role in the development of ischemic stroke and related conditions. In experimental models, inhibiting necroptosis has been shown to reduce brain infarct size and improve motor and cognitive outcomes, indicating its potential neuroprotective value. Another form of programmed cell death, pyroptosis, is characterized by rapid plasma membrane rupture and the release of proinflammatory contents, further contributing to post-ischemic inflammation (Shu et al., 2022).

Autophagy is a cellular process in which cytoplasmic proteins or organelles are engulfed into vesicles that fuse with lysosomes to form autolysosomes, where the contents are subsequently degraded (Mo et al., 2020). Autophagy acts as a double-edged sword, capable of either repairing or degrading damaged neurons following an ischemic insult. However, excessive or prolonged activation of autophagy can lead to neuron damage and trigger cell death (Ajoolabady et al., 2021).

### 3.5 Ferroptosis

Iron is involved in several mechanisms of neuronal damage following ischemic stroke. It plays a key role in major molecular processes like free radical production, excitotoxicity, and neuroinflammation. Additionally, its accumulation can lead to ferroptosis (Guo et al., 2023). Existing research shows that ferroptosis regulation is primarily governed by the control of iron, lipid peroxidation, and several antioxidant systems, including the GSH/GPX4 axis, CoQ10/FSP1 axis, and others (Xu et al., 2023). Glutathione (Glu-Cyc-Gly), a tripeptide, helps protect cells from oxidative damage by interacting with free radicals. When glutathione (GSH) is converted to oxidized glutathione (GSSG), the antioxidant enzyme Gpx4 reduces harmful lipid peroxides to alcohols. During ferroptosis, redox-active iron accumulates through the Fenton reaction, depleting GSH reserves, inhibiting Gpx4, and triggering an excessive antioxidant response (Liu et al., 2020). Ferroptosis, a unique form of programmed cell death (PCD), is characterized by extreme iron overload and lipid peroxidation driven by ROS production. This pathway plays a crucial role in neuron death. Unlike other forms of cell death, ferroptosis is marked by the reduction or loss of mitochondrial cristae and a condensed mitochondrial membrane structure (Wang et al., 2020). Ferroptosis is characterized by distinct morphological changes in mitochondria, such as fragmentation, reduced size, rupture of the outer mitochondrial membrane (OMM), and the disappearance of mitochondrial cristae (She et al., 2023). Ferritin-rich brain tissue and iron-rich blood are the result of direct compression and stimulation of the hematoma, which weakens the blood-brain barrier and causes primary brain injury (Mo et al., 2020). The release of iron from the hematoma after intracerebral hemorrhage (ICH) can trigger perihematomal edema, oxidative stress, and increased ROS levels, ultimately leading to ferroptosis (Dong et al., 2023).

## 4 Traditional medicines in stroke management

### 4.1 Traditional Chinese medicine

Traditional Chinese medicine has been utilised to prevent various illnesses in Asian nations, particularly China. Chinese Materia medica, TCM preparation, and their active metabolite(s) may help treat brain damage brought on by ischemia/reperfusion (Table 1). Numerous studies on TCM’s ability to protect the BBB have been published in recent years (Li et al., 2019).The botanical drug(s) resistance to platelets, such as *Toxicodendron vernicifluum* (Anacardiaceae), *Salvia miltiorrhiza* Bunge (Lamiaceae), *Biancaea sappan*. (Fabaceae), *Curcuma aromatica* Salisb.(Zingiberaceae)*,CinnamomumcassiaPresl* (Lauraceae),*Paeonialactiflora* (Paeoniaceae),PanaxginsengC.A.Mey.(Araliaceae),*Carthamus tinctoriusa* (Asteraceae) (Kim and Park, 2019). Traditional Chinese medicine (TCM) offers several notable advantages in treating multisite, multitarget disorders and general regulation. Certain TCMS, including *Gastrodia elata* (Orchidaceae), *Rehmannia glutinosa* (Orobanchaceae), *Ginkgo biloba* (Ginkgoaceae), and *Panax notoginseng* (Araliaceae), show higher therapeutic effects on neurological illnesses than on other conditions. NBP, or dl-3-n-butylphthalide, has been shown to possess neuroprotective properties (Zhu et al., 2021). Phenolic glycosides are characteristic of Salidroside, which is obtained from the traditional Tibetan medicinal plant *Rhodiola rosea* (Crassulaceae). Traditional Chinese medicine is commonly used to increase the body’s resistance to weariness. Numerous studies have demonstrated that it has various biological effects, including antioxidant properties (Zuo et al., 2018).

**TABLE 1:** Chinese herbal medicines and their outcomes.

S.no	Herbal medicines	Key metabolite(s)	Mechanism of action	Reference
1	Bu-yang-huan-wu-tang	*Angelica sinensis* (Oliv.) Diels (Apiaceae), *Astragalus mongholicus* Bunge Fabaceae), *Conioselinum anthriscoides* ‘Chuanxiong’ (Apiaceae), *Carthamus tinctorius* L. (Asteraceae), *Carthamus tinctorius* L., Prunus persica (L) Batsch (Rosaceae), and *Paeonia lactiflora* Pall (Paeoniaceae)	Neuroprotection: BYHWT supports neuronal survival by reducing oxidative stress, preventing apoptosis, and improving mitochondrial function. Anti-inflammatory Effects: It also modulates inflammatory pathways, lowering pro-inflammatory cytokine levels and promoting neurovascular repair	Hung et al. (2015)
2	Danhong injection	*Salvia miltiorrhiza* Bunge [Lamiaceae] and *Carthamus tinctorius* L. [Asteraceae]	These processes help upregulate the expression of growth-associated protein 43 (GAP-43), promote axonal regeneration, and accelerate neural functional recovery	Han et al. (2017)
3	Sanhua	*Rheum officinale* Baill. [Polygonaceae], *Magnolia officinalis Rehder* & E.H.Wilson [Magnoliaceae], *Citrus aurantium* L. [Rutaceae], and *Hansenia weberbaueriana* [Apiaceae]	SHD significantly improved rheological parameters, including blood viscosity, fibrinogen levels, and haematocrit, thereby enhancing blood flow and reducing the risk of vascular complications	Zheng et al. (2023)
4	Zhi-gan-cao-tang	*Glycyrrhiza glabra* L. [Fabaceae],*Panax ginseng* C.A.Mey. [Araliaceae], *Neolitsea cassia* (L.) Kosterm. [Lauraceae], *Zingiber officinale* Roscoe [Zingiberaceae], *Ophiopogon japonicus* (Thunb.) Ker Gawl. [Asparagaceae], *Ziziphus jujuba* Mill. [Rhamnaceae], *Rehmannia glutinosa* (Gaertn.) Libosch. Ex DC. [Orobanchaceae], Cannabis sativa L. [Cannabaceae]	ZGCT may assist individuals with premature ventricular contractions by alleviating associated symptoms such as heart palpitations, shortness of breath (dyspnea), insomnia, and fatigue	Hung et al. (2016)
5	Ginkgo biloba leaf	*Ginkgo biloba* L. [Ginkgoaceae]	*Ginkgo biloba* extract (GBE) is developed to optimize the therapeutic potential of its leaves, with EGb761 being the standardized formulation. In traditional Chinese medicine, Ginkgo biloba leaves are believed to astringe the lungs, regulate the intestines, promote blood circulation, and nourish the heart	Zhao et al. (2021a)
6	Huanglian Jie Du decoction	*Coptis chinensis* Franch. [Ranunculaceae], *Scutellaria baicalensis* Georgi [Lamiaceae], *Phellodendron amurense* Rupr. [Rutaceae], *Gardenia jasminoides* J. Ellis [Rubiaceae]	Both extrinsic pathways, like death receptor-mediated apoptosis, and intrinsic pathways, such as those mediated by mitochondria, calpain, p53, p38, ERK, JNK, and the endoplasmic reticulum, are crucial for activating downstream apoptotic molecules that lead to cell death	Yu et al. (2022)
7	Buyang Huanwu	*Astragalus mongholicus* Bunge [Fabaceae] *Prunus persica* (L.) Batsch [Rosaceae], *Carthamus tinctorius* L. [Asteraceae] *Paeonia lactiflora* Pall. [Paeoniaceae], *Angelica sinensis* (Oliv.) Diels [Apiaceae] *Conioselinum anthriscoides* ‘Chuanxiong’ [Apiaceae]	By activating the PI3K-Akt signaling pathway, BHD may disrupt the progression of Ischemic stroke (IS) and induce cuproptosis, thereby exerting anti-apoptotic effects	Zhong et al. (2023)
8	Angong Niuhuang pill	*Bos taurus domesticus* Curcuma aromatica Salisb. [Zingiberaceae] *Rhinoceros unicornis* Gardenia jasminoides J. Ellis [Rubiaceae] Coptis chinensis Franch. [Ranunculaceae] *Scutellaria baicalensis* Georgi [Lamiaceae]	In brain tissue, *Angong Niuhuang* Pill has been shown to significantly reduce NO levels and NOS activity. Additionally, it can increase the erythrocyte aggregation index and platelet aggregation rate, while enhancing both blood and plasma viscosity	Xu et al. (2022)
9	Qingkailing (QKL) injection	*Isatis tinctoria* L. [Brassicaceae] *Lonicera japonica* Thunb. [Caprifoliaceae] *Concha Margaritifera* Usta, *Betula pubescens* var. pubescens [Betulaceae] *Gardenia jasminoides* J.Ellis [Rubiaceae], and *Cornus mas* L. [Cornaceae]	In a mouse model of cerebral ischemia/reperfusion, QKL injection has been shown to reduce calcium overload, enhance endothelial nitric oxide synthase expression, regulate matrix metalloproteinase-9 levels, and suppress inflammatory responses	Cheng et al. (2012)
10	Xue-Fu-Zhu-Yu Decoction (XFZYD)	*Angelica sinensis* (Oliv.) Diels (Apiaceae) Root, *Bupleurum chinensis* (Apiaceae)*, Carthamus tinctorius* L. (Asteraceae)*, Citrus limon (L.) Osbeck* (Rutaceae), *Cyathula officinalis* K.C. Kuan (Amaranthaceae), *Glycyrrhiza glabra L*. (Leguminosae), *Conioselinum anthriscoides* ‘Chuanxiong’ (Apiaceae), *Paeonia lactiflora* Pall. (Paeoniaceae), *Platycodon grandiflorus* (Jacq.) A. DC. (Campanulaceae), *Prunus persica* (L.) Batsch (Rosaceae), *Rehmannia glutinosa* (Gaertn.) Libosch. Ex DC. (Orobanchaceae)	XFZYD enhanced rt-PA-mediated neuroprotection in rats with thromboembolic stroke. This effect is likely due to the suppression of HIF-1α and TNF-α, which in turn inhibits inflammation (via iNOS) and apoptosis (via active caspase-3). These findings provide scientific support for the therapeutic potential of combining XFZYD with rt-PA in the treatment of Ischemic stroke	Lee et al. (2011)

Composition of the traditional Chinese medicine of some remedies used for ischemic stroke. The botanical herbs used for the preparation of Xue-Fu-Zhu-Yu decoction and the Amount used are *Angelica sinensis* (Oliv) Diels (Apiaceae) Root 4.5 g, *Bupleurum chinensis* (Apiaceae) Root 1.5 g*, Carthamus tinctorius* L. (Asteraceae) Flower 4.5 g*, Citrus × limon (L.) Osbeck* (Rutaceae) Fruit 3.0 g, *Cyathula officinalis* K.C.Kuan (Amaranthaceae) Root 4.5 g, *Glycyrrhiza glabra L*. (Leguminosae) Root 1.5 g, *Conioselinum anthriscoides* ‘Chuanxiong’ (Apiaceae) Root 2.3 g, *Paeonia lactiflora* Pall. (Paeoniaceae) Root 3.0 g, *Platycodon grandiflorus* (Jacq.) A. DC. (Campanulaceae) Root 2.3 g, *Prunus persica* (L.). Batsch (Rosaceae) Seed 6.0 g, *R. glutinosa* (Gaertn.) Libosch. ex DC. (Orobanchaceae) Root 4 g (Lee et al., 2011). *Buyang Huanwu* decoction consist of seven botanical herbs such as *A. sinensis* (Oliv.) Diels (Apiaceae), *Astragalus mongholicus* Bunge (Fabaceae) (120 g) dried roots, *C. anthriscoides* “Chuanxiong:” (Apiaceae) 3 g dried rhizomes, *C. tinctorius* L. (Asteraceae) 3 g dried flowers *of C. tinctorius* L., Prunus persica (L) Batsch (Rosaceae) 3 g dried seeds, and *P. lactiflora* Pall (Paeoniaceae) 4.5 g dried roots (Zhang et al., 2018).

Notably, Table 2 summarizes several *in vivo* studies showcasing the molecular and functional outcomes of these TCMs, supporting their potential as adjunct therapies in ischemic stroke treatment.

**TABLE 2:** *In vivo* studies of traditional Chinese medicines used in Ischemia stroke.

S.no	Background	Methodology	Molecular findings	Conclusion	Ref
1	EGb 761, a standardized extract of Ginkgo biloba, exhibits neuroprotective effects in ischemic stroke. In a rat stroke model, MRI has been used for non-invasive evaluation of its influence on neurovascular reconstruction and axonal regeneration	Male Sprague-Dawley rats underwent permanent right middle cerebral artery occlusion (MCAO) and were treated daily with either EGb 761 (60 mg/kg) or saline for 15 days, starting 6 h post-MCAO. Functional recovery was assessed using beam walking. Structural and vascular changes were evaluated through multi-parametric MRI, histology, and Western blot analysis of remodeling-related proteins	Treatment increased p-AKT and p-GSK-3β levels, decreased p-CRMP2, and upregulated GAP-43. Additionally, it downregulated the axonal growth inhibitors NogoA and NgR	EGb 761 facilitated neurovascular restoration, enhanced endogenous neurogenesis, and supported axonal regeneration, all of which contributed to both structural and functional recovery after a stroke	Li et al. (2018)
2	EGb 761 is a standardized extract of Ginkgo biloba containing 24% ginkgo-flavonol glycosides and 6% terpene lactones. Known for its neuroprotective effects, the mechanisms behind its action, particularly those involving heme oxygenase 1 (HO1), are not well understood. This research aimed to investigate the neuroprotective potential of EGb 761 and its bioactive metabolites—Bilobalide (BB), Ginkgolide A (GA), Ginkgolide B (GB), and Terpene Free Material (TFM)—under ischemic conditions	Mice underwent permanent distal middle cerebral artery occlusion (pMCAO) and were observed for 7 days. Both HO1 knockout and wildtype (WT) mice were used to investigate the role of HO1. Treatment with EGb 761 or compounds was administered 4 h following pMCAO.	Protein levels of HO1, VEGF, and eNOS were increased in the EGb 761 and BB-treated groups, but not in the GA, GB, or TFM-treated groups, highlighting the specific role of these compounds in neurovascular protection	The HO1-induced pathway plays a key role in mediating the neuroprotective effects of EGb 761. Identifying target molecules that are elevated by natural substances provides a novel approach to enhancing stroke treatment	Shah et al. (2011)
3	The protective action of Xue-Fu-Zhu-Yu Decoction (XFZYD), either alone or in combination with recombinant tissue plasminogen activator (rt-PA), was assessed in a rat model of thromboembolic stroke. XFZYD, a traditional cardiovascular disease remedy, has been shown to promote endothelial progenitor cell angiogenesis and may be used as adjunct therapy for ischemic stroke	A rat model of cerebral thromboembolic stroke was used to assess the effects of interventions: rt-PA (8 mg/kg), XFZYD (1.5 or 3.0 g/kg/day), either alone or in combination. The outcomes were evaluated by measuring infarct volume and analyzing protein expression (TNF-α, iNOS, HIF-1, active caspase-3) through immunoblotting	XFZYD enhanced rt-PA-mediated suppression of inflammatory markers (TNF-α, iNOS) and apoptotic markers (HIF-1, caspase-3), indicating a synergistic neuroprotective mechanism	XFZYD significantly enhances rt-PA-mediated neuroprotection in thromboembolic stroke, likely by inhibiting inflammation and apoptosis. These results support the potential of XFZYD as an adjunct therapy to rt-PA for ischemic stroke treatment	(Lee et al., 2011).
4	Investigate the expression of transforming growth factor-β1 (TGF-β1) in rats with cerebral ischemia-reperfusion injury following treatment with Qingkailing injection (QKL)	Three groups of healthy Wistar rats were randomly assigned: QKL-treated, normal, and model (ischemia-reperfusion). Bilateral common carotid artery (CCA) occlusion was performed to induce cerebral ischemia-reperfusion injury. Immunohistochemistry was used to assess the expression of TGF-β1 and glial fibrillary acidic protein (GFAP) in brain regions at various time points	Following ischemia-reperfusion injury, both GFAP and TGF-β1 expression levels were elevated. However, treatment with QKL significantly reduced the expression of GFAP and TGF-β1 at various time points compared to the untreated model group	QKL may exert neuroprotective effects in cerebral ischemia-reperfusion injury by downregulating TGF-β1 expression, potentially through the inhibition of astrocyte (AST) activation	Yan-shu et al. (2008)
5	Investigation of the therapeutic effect and underlying mechanisms of Ruyi Zhenbao pill in promoting neurological recovery following cerebral ischemia/reperfusion injury in rats	Male Sprague-Dawley rats underwent middle cerebral artery occlusion (MCAO) followed by reperfusion. The rats were treated intragastrically with Ruyi Zhenbao pill at doses of 0.2, 0.4, or 0.8 g/kg for 14 days. Neurological function was assessed through cylinder, adhesive removal, and beam-walking tests. Neurogenesis and angiogenesis were evaluated via immunofluorescence staining, while levels of BDNF, NGF, and VEGF were measured using ELISA.	The beneficial effects of Ruyi Zhenbao are linked to the enhanced expression of neurotrophic and angiogenic factors, such as BDNF, NGF, and VEGF, which promote improved neurogenesis and angiogenesis	Ruyi Zhenbao pill aids neurological recovery following cerebral ischemia/reperfusion by stimulating neurogenesis and angiogenesis, primarily through the upregulation of BDNF, NGF, and VEGF.	WANG et al. (2016)
6	Investigation of the neuroprotective mechanisms of Huang-Lian Jie-Du Decoction (HLJDD) on cerebral ischemia involves understanding its metabolic targets and pathways through a systems biology approach	Cerebral ischemia was induced in rats using the middle cerebral artery occlusion (MCAO) model with reperfusion. The pharmacological effects of HLJDD were tested, and plasma extracts from control, MCAO, and HLJDD-treated rats were analyzed using UPLC-Q-TOF/MS. Data processing was done with MassLynx and EZinfo 2.0, followed by analysis through PCA, OPLS-DA, and pathway databases (HMDB, KEGG, Metlin, SMPD). Biochemical assays on brain tissue were performed to confirm key pathways	HLJDD’s therapeutic effects are based on its ability to modulate multiple targets, influencing key neurochemical processes such as energy metabolism, neurotransmitter balance, and neuroinflammation	HLJDD exerts neuroprotective effects in cerebral ischemia by modulating metabolic stress, regulating glutamate metabolism, and enhancing acetylcholine function, thereby highlighting its multi-target, systems-level action	Zhu et al. (2018)
7	The neuroprotective mechanism of Buyang Huanwu Decoction (BHD) in treating ischemic stroke is investigated, with a specific focus on the S1P/S1PR1/PI3K/Akt signaling pathway	A chemically analyzed lyophilized form of BHD was investigated using UPLC-Q-TOF/MS. A mouse model of permanent middle cerebral artery occlusion (pMCAO) was established. Male KM mice were divided into seven groups: sham, model, FTY720, BHD, BHD + W146 (S1PR1 inhibitor), SEW2871 (S1PR1 agonist), and Calycosin. Treatments were administered for 14 days. Outcomes included neurological scores (mNSS), infarct volume, and protein/mRNA expression of SphK1, SphK2, S1PR1, PI3K, Akt, and p-Akt, which were assessed by Western blot, IHC, and qRT-PCR in brain areas	SEW2871 (agonist) mimicked BHD’s effects by enhancing the expression of S1PR1, Akt, and SphK2. The neuroprotective effects of BHD are likely mediated through the activation of the S1P/S1PR1/PI3K/Akt pathway and the inhibition of SphK1	BHD protects against cerebral ischemic injury by modulating the S1P/S1PR1/PI3K/Akt signaling pathway, reducing SphK1 levels, and increasing SphK2 along with downstream protective proteins. This supports BHD as a potential treatment for ischemic stroke	Liu et al. (2023)

### 4.2 Herbal medicines

“In the last 30 years, polyphenols and micronutrients in foods have gained attention for their antioxidant effects and potential to prevent diseases like cancer, heart problems, and brain disorders linked to oxidative stress. Polyphenols, made by plants, help protect against UV rays and infections. People consume about 1 g of polyphenols daily, much more than vitamin E or C, making them a major source of dietary antioxidants (Pacifici et al., 2021). Polyphenols are plant compounds classified into five groups: lignans, curcumins, stilbenes, phenolic acids, and flavonoids. In stroke models, they show neuroprotective effects by reducing brain damage and aiding recovery. Their antioxidant properties come from their ability to bind metals and neutralize reactive oxygen species. Key polyphenols like gallic acid, resveratrol, and quercetin help protect against ischemic stroke by targeting multiple pathways involved in damage and repair (Abdelsalam et al., 2023). Polyphenols have gained attention for their health benefits, especially their antioxidant and neuroprotective effects after ischemic stroke, as shown in recent studies (Zhou et al., 2021).

#### 4.2.1 Resveratrol

Resveratrol (3,5,4′-trihydroxy-trans-diphenyl-ethylene) is a natural antioxidant that helps combat inflammation and neutralize free radicals in the body (Fodor et al., 2018). Resveratrol promotes neuroprotection in ischemic stroke by activating SIRT1 and NRF2 pathways. This enhances mitochondrial function, antioxidant defense, and stress response, helping cells survive and reducing apoptosis (Owjfard et al., 2024).

#### 4.2.2 Gallic acid

Gallic acid is a well-absorbed polyphenol that helps reduce cerebral edema, a common issue after ischemic stroke. It also promotes microglial activation and shifts their phenotype, aiding in stroke recovery (Qu et al., 2022). Mechanistically, GA has been demonstrated to control M1-type macrophage polarization processes and, in part, suppress inflammatory responses by preventing M1-type macrophage polarization. Furthermore, it has been demonstrated that GA–chitosan complexes alter inflammatory responses in lipopolysaccharide-stimulated RAW264.7 cells (macrophage line) via affecting the cellular NF-κB, AP-1, and MAPK pathways (Qu et al., 2022).

#### 4.2.3 Curcumin

One of the primary active metabolite(s) in Chinese botanical drug(s), curcumin, regulates the NLRP3 signalling pathway to promote neuronal repair and neuroprotection (Du et al., 2023).According to a study, pretreatment with curcumin reduces neuronal mortality in the CA1 region of the hippocampus of rats with LPS-induced depression, which is accompanied by an improvement in synaptic function (Fan et al., 2021).Additionally, curcumin therapy improved cell survival, reduced cell apoptosis, and increased Bcl-2 protein levels while decreasing caspase-3 expressions and Bax in mouse N2a cells after OGD/R injury. Curcumin treatment also stopped Bax activation and maintained the integrity of the mitochondrial membrane (Xie et al., 2018).

#### 4.2.4 Flavonoid

Flavonoids, which are abundant in fruits and vegetables and other foods we eat every day, have been proven to have positive therapeutic effects on cerebral ischemia injury by reducing neuroinflammation. Apigenin, baicalein, Naringenin, EGCG, quercetin, rutin, and other flavonoids, for instance, have been found to inhibit and limit neuroinflammation following ischemic stroke by affecting the activation of astrocytes and microglia (Lu et al., 2024).Flavonoids are classified into six groups based on the various structures that link the two benzene rings: flavanols, flavanones, flavonols, isoflavones, flavones, and anthocyanidins (Liu S. et al., 2022).The degree of unsaturation, the location of the benzoid substituent, and the orientation of hydroxylation or methylation are some characteristics that distinguish different flavonoids from one another. Their antiproliferative and antioxidant properties are limited by their structural features (Cebova and Pechanova, 2020).The neuroprotective properties of flavonoids are found in Chinese herbal medicine. By reducing excitotoxicity, oxidative stress, inflammation, thrombin toxicity, and cell deaths, as well as by preserving the blood-brain barrier, Ca_2_^+^ overloading and neurogenesis, flavonoids, a type of botanical medication, have a neuroprotective effect (Zhou et al., 2024).

#### 4.2.5 Quercetin

Quercetin reduces blood-brain interference and condenses the quantity of MMP-9 in tests for cerebral ischemia, according to antioxidant analyses that are similar to those of green tea polyphenols. By reducing lipid peroxidation and ion channel acid sensing, two factors that contribute to ion channel dysregulation, quercetin helps prevent ischemic injury (Maqbool and Zehravi, 2021). Quercetin prevents platelet aggregation by inhibiting agonists like thrombin, collagen, and ADP. It also blocks calcium signaling and binds to the GPIIb/IIIa receptor to reduce platelet activity. In C57BL/6 rats, this action helps reduce thrombosis and improves blood flow after FeCl_3_-induced carotid artery injury (Zhang et al., 2022). Table 3 illustrates *In vivo* studies performed in the ischemic stroke using herbal medicines.

**TABLE 3:** *In vivo* studies performed for ischemia stroke using herbal medicines.

Natural compound	Animal model & treatment	Dosage & duration	Outcomes & mechanisms	Reference
Astragalosides	MCAO in rats	10–50 mg/kg, i.p., daily for 7 days	Reduced infarct size, anti-inflammatory, antioxidant, neuroprotection	Shang et al. (2022)
Baicalin	MCAO in rats	50–100 mg/kg, oral, 14 days	Reduced apoptosis, oxidative stress, improved BBB integrity	fan et al. (2025)
Glycyrrhizin	MCAO in rats	10–20 mg/kg, i.p., daily for 5 days	Suppressed HMGB1, reduced neuroinflammation, decreased infarct size	Li et al. (2024)
Ginseng	MCAO in mice	100–200 mg/kg, oral, 7 days	Enhanced neurogenesis, reduced oxidative damage, improved motor function	Liu et al. (2019)
Curcumin	MCAO in rats	80 mg/kg, oral, 10 days	Antioxidant, anti-inflammatory, reduced infarct volume	Gokce et al. (2016)
Resveratrol	MCAO in mice	20 mg/kg, i.p., daily for 14 days	Increased SIRT1 expression, reduced oxidative stress	Lopez et al. (2015)
Quercetin	MCAO in rats	50 mg/kg, oral, 7 days	Anti-inflammatory, modulated Nrf2 pathway, reduced neuronal death	Zhang et al. (2022)
Epigallocatechin Gallate (EGCG)	MCAO in mice	30 mg/kg, oral, 14 days	Reduced neuroinflammation, enhanced antioxidant defense	Sun et al. (2023)
Hesperidin	MCAO in rats	50 mg/kg, oral, 7 days	Attenuated oxidative stress, reduced BBB disruption	Lee et al. (2020a)
Berberine	MCAO in rats	100 mg/kg, oral, 14 days	Anti-inflammatory, improved mitochondrial function	Zhao et al. (2021b)

### 4.3 Ayurveda

The oldest and most significant tradition in India, Ayurveda, is founded on experimentation and philosophy. With an emphasis on strengthening the host’s immune system, Ayurveda has a database of numerous medicinal botanical drug(s) and is accessible in several regional languages. According to several classical books, Ayurveda mentions 1200 illnesses. The 700 therapeutic botanical drug(s) in the Atharveda (c. 1200 BC), Charak Samhita, and Sushrat Samhita (c. 1000–500 BC) (Ara et al., 2022).*Adoxa moschatellina, Bacopa monnieri, Centella asiatica, Mucuna urens, Phyllanthus emblica, Terminalia arjuna, and Withania somnifera* are the plants that are the basis for the Mentat, also known as BR-16A. Because of its free-radical-scavenging qualities and antioxidant, it functions as a neuroprotective agent and may be utilized to help patients recover from ischemic stroke (Ibáñez et al., 2023). Ashwagandha (*W. somnifera)* is a traditional, well-known Indian medicinal plant that is frequently used as a component in Ayurvedic formulations sold to treat neurological conditions. It is helpful. Inhibition of gelatinases (MMP-9) Therapeutically active in ischemic stroke and hemorrhagic stroke (Zahiruddin et al., 2020).Ayurveda views hemiplegia as a clinical entity, “*Pakshaghata*,*”* grouped under “*Vatavyadhi.”* The central role of Ayurveda lies during the time of stroke rehabilitation. It employs internal medicines along with procedures, including *Panchakarma* therapies. Evidence-based medicine (EBM) requires proof of effectiveness, efficiency, and safety for decision-making in patient care. Integrating individual clinical expertise with the best external clinical evidence is the core of EBM practice. Thus, empirical Ayurvedic knowledge accrued over the years can be tapped for integrated stroke rehabilitation after proper evaluation (M. A et al., 2025).

## 5 Mechanisms of action involved in TCIM approaches

Studies show that acupuncture combined with the Tianma Gouteng Yin formula is effective in treating acute cerebral infarction. This combination significantly improves vascular endothelial function and cerebral vascular function, including measures like pulsatility index, cerebrovascular reserve, neurological function, platelet activation, and inflammation (IL-6, hs-CRP). It also positively impacts NIHSS and BI scores (Zhang et al., 2023). TCM can treat stroke through multiple targets and pathways with lower side effects. It affects pathways like AKT/PI3K and SLC7A11/GPX4, which promote angiogenesis, neuroprotection, and reduce ferroptosis. This multi-target approach may lead to more effective stroke treatment (Fu et al., 2022). A study found that Astragaloside IV could boost the levels of SLC7A11 by activating the Nrf2/HO-1 signalling pathway. and GPX4 and ROS. In an ICH model with intravascular perforation, this activation resulted in increased antioxidant capacity and lipid peroxidation inhibition (Liu Z. et al., 2022). Through several processes, TCM has been utilized to treat stroke either alone or in conjunction with other treatments. such include promoting neurogenesis and angiogenesis, regulating the blood-brain barrier, inhibiting platelet activation, preventing inflammation, oxidative stress, and apoptosis (Chen and Jin, 2023).

## 6 Complementary medicines

Complementary alternative medicine encompasses a variety of goods, techniques, and structures that are not typically associated with traditional care. Geographical and cultural disparities may cause different CAMs to be utilized in Asian and African nations to treat stroke. Over the past 10 years, stroke patients’ interest in complementary and alternative medicine has rapidly increased (Chang et al., 2016). Integrative medicine addresses patients’ complicated demands by combining alternative therapies with traditional medical treatments, especially in cases of ischemic stroke. Nowadays, a lot of people use Chinese medicine in conjunction with conventional therapy to prevent and cure stroke (Ni et al., 2020).

### 6.1 Yoga

Yoga therapy is based on the Panchakosha concept from the Taittiriya Upanishad, which views a person as having five layers: physical (Annamaya), energy or life force (Pranamaya), mind (Manomaya), intellect (Vijnanamaya), and bliss (Anandamaya) (Jasti et al., 2022). Yoga combines breathing, stretching, and balance to improve both mental and physical wellbeing. It helps calm the mind and build focus. Breathing is coordinated with various poses, whether standing, sitting, lying, or prone (Pal et al., 2023). Yoga may help control key risk factors for stroke, especially high blood pressure—the most modifiable risk factor. Evidence suggests it can safely and effectively reduce hypertension in stroke patients (Thayabaranathan et al., 2018). Yoga boosts hemoglobin and red blood cell levels, improving oxygen delivery to cells. It also helps lower blood pressure, reducing the risk of stroke and heart failure caused by blood clots (Dubey, 2024). In a pilot study by Arlene et al., 47 chronic stroke patients were randomized to either therapeutic yoga (n = 37) or a control group (n = 10). After 8 weeks, the yoga group showed significant improvements in physical function. This is important, as chronic stroke often leads to disabilities that affect daily activities and social participation (Schmid et al., 2014).

### 6.2 Acupuncture

Acupuncture, a key part of Traditional Chinese Medicine, involves inserting fine needles into specific body areas (Mu et al., 2023). Acupuncture has been used to treat stroke since ancient times, including body, scalp, eye, and electro-acupuncture methods. Choosing the correct points is crucial for effective treatment (Li et al., 2022). Acupuncture supports neurogenesis after ischemic injury by boosting neural stem cell activity and reducing local inhibitory factors, creating a healing-friendly environment (Mu et al., 2023). Current studies suggest that acupuncture may aid in treating ischemic stroke by reducing oxidative stress, protecting the blood-brain barrier, and regulating exosomes involved in neuroprotection, neuroplasticity, cell growth, apoptosis, immunity, and inflammation (Zhu et al., 2024).

#### 6.2.1 Mechanism of action

Acupuncture may help reduce inflammation in ischemic stroke by lowering proinflammatory cytokines like IL-1β, IL-6, and TNF-α in the brain and blood. This effect is likely linked to inhibition of NF-κB activation, which reduces cytokine production (Kuang et al., 2024). The following sections discuss methods to prevent oxidative stress, including repairing damaged proteins, lipids, or DNA, limiting ROS-induced cell death or autophagy, reducing ROS production, and neutralizing ROS through antioxidant enzymes or other signaling pathways (Poljsak, 2011).

Acupuncture and low-intensity laser (LA) therapy are used to treat various conditions. In rats with right middle cerebral artery blockage, LA at GV20 reduced MDA levels and increased the activity of GPx, CAT in the cerebral cortex, and SOD in mitochondria (Jittiwat, 2017). A randomized controlled trial assessed acupuncture’s effectiveness in ischemic stroke rehabilitation. Patients were divided into two acupuncture groups and one control group with only rehabilitative instructions. After 2 weeks, the acupuncture groups showed significant improvements in NIHSS scores, and the second acupuncture group had higher BI scores than the control. These results suggest that acupuncture may aid in functional rehabilitation post-stroke (Li et al., 2022).

### 6.3 Physical exercise

Exercise promotes neuroplasticity, neurotrophins, and cognitive function, while improving overall health and reducing the risk of hypokinetic diseases associated with a sedentary lifestyle (Sakakima, 2019). After a stroke, physical therapy aids in regaining function and movement. While task-specific training was once the main focus, recent attention has shifted to exercise-based rehabilitation (Pogrebnoy and Dennett, 2020). Exercise after a stroke may enhance the expression of Ang-1 and Tie-2, aiding brain recovery. Ischemic stroke can impair memory by increasing apoptosis and affecting BDNF levels in the hippocampus. Treadmill training helps recover memory by boosting BDNF expression, promoting cell growth, and reducing apoptosis (Xing et al., 2018). Cerebral ischemia triggers a cascade of events that increase cerebrovascular permeability and disrupt the blood-brain barrier (BBB), leading to brain edema. MMP-9 contributes to BBB failure by breaking down the extracellular matrix (Zhang et al., 2019). Exercise preconditioning strengthens the basal lamina and reduces blood-brain barrier (BBB) dysfunction in ischemic stroke by increasing MMP-9. Chronic cerebral hypoperfusion can be countered by treadmill exercise, which boosts MMP-9 levels, lowers occludin, and degrades zonula occludens-1 (ZO-1) (Lee SS. et al., 2020).

### 6.4 Dietary and nutritional supplementation

Nutrition plays a key role in stroke risk, which can be reduced by up to 80% with a healthy lifestyle, also lowering stroke risk factors (Spence, 2019). The former treatment regimen involves consuming specific nutrients (e.g., metals, fibers, fatty acids, vitamins), while the latter refers to time-restricted feeding, fasting, or nutrient restriction (e.g., carbs, amino acids). Both approaches, used in preclinical or clinical settings, help maintain nutritional balance and may slow the progression of neurological diseases (Mao et al., 2021). The former treatment regimen involves consuming specific nutrients (e.g., metals, fibers, fatty acids, vitamins), while the latter refers to time-restricted feeding, fasting, or nutrient restriction (e.g., carbs, amino acids). Both approaches, used in preclinical or clinical settings, help maintain nutritional balance and may slow the progression of neurological diseases (Ciancarelli et al., 2024). Research shows that omega-3 fatty acids, found in fish, nut oils, and leafy vegetables, improve neurotransmission and prevent membrane fluidity loss caused by cholesterol. These effects help maintain cell signaling and synaptic function. Regular intake of polyphenol-rich foods can enhance cerebral blood flow, cognition, and promote neurogenesis and synaptogenesis. Flavonoids, present in many edible plants, may support neuroplasticity, neurogenesis, and cerebral blood flow, thanks to their anti-inflammatory and antioxidant properties (Siotto et al., 2022).

#### 6.4.1 Vitamins

Vitamins and minerals can support neuroprotection and recovery by boosting antioxidant capacity and aiding functional recovery in stroke patients. Antioxidants like vitamin E and C help reduce oxidative damage (Lieber et al., 2018). Vitamin C administration improves microcirculation, neurological outcomes, and survival, while reducing oxidative stress, heart damage, and arrhythmias (Spoelstra-de Man et al., 2018). The study showed that supplementing with tetrahydrobiopterin, arginine, and vitamin C improved blood flow during ischemia by reducing oxidative stress and increasing endothelial NO synthase activity (Tang et al., 2022). Vitamin D may have antithrombotic effects by reducing cytokine-induced changes in tissue factor and thrombomodulin, as suggested by several experimental studies (Cui et al., 2024). EPA metabolites from Omega-3 fatty acids reduce inflammation, lower monocyte adhesion and platelet aggregation, and improve endothelial function by inhibiting COX-1/2 enzymes, leading to less production of inflammatory prostaglandins (Ueno et al., 2019).

#### 6.4.2 Minerals

Copper (Cu) and zinc (Zn) are the most common metals in the human body and are found in high concentrations in the brain. While their exact roles in inflammation are still being studied, they are essential for controlling oxidative stress and regulating the production of free radicals (Wessels et al., 2017). Selenium (Se) is an essential mineral with roles in immune function, cancer prevention, and cell regulation. As part of the antioxidant system, selenium and its proteins may also offer neuroprotective benefits (Mirończuk et al., 2021).

### 6.5 Aromatherapy

Essential oils are used therapeutically in aromatherapy as a supplemental medicine to cure a variety of mental and physical illnesses and to advance patient wellbeing (Reis and Jones, 2017). Essential oil components can enter the bloodstream and cross the blood-brain barrier through various routes such as topical, subcutaneous, oral, intraperitoneal, or inhalation applications. Aromatic oils like lavender, bergamot, and curcuma have shown numerous beneficial effects (Contrada et al., 2021). It has been demonstrated that essential oils contain anti-inflammatory and antioxidant qualities in addition to psychological benefits that might lessen emotional and physical ailments like exhilaration, exhaustion, and delirium (Lim and Kim, 2024). Neurotransmitter systems involved in neuronal survival and plasticity are influenced by specific essential oils. For example, it has been demonstrated that chemicals in sandalwood oil interact with gamma-aminobutyric acid (GABA) receptors, modifying excitatory and inhibitory neurotransmission to promote neuroprotective effects (Younis and Mohamed, 2020). Essential oils like bergamot and lavender, known for their antioxidant properties, help reduce oxidative stress during ischemia. They boost antioxidant enzymes like glutathione peroxidase and superoxide dismutase, which lower ROS production and cellular damage. Additionally, these oils have anti-inflammatory effects by suppressing pro-inflammatory cytokines, reducing inflammation linked to ischemic stroke (Contrada et al., 2021).

### 6.6 Music therapy

Music therapy uses musical elements to improve mood and neurological function. It has shown positive results in stroke rehabilitation across various studies (Xu et al., 2022). A home-based music therapy program was developed for self-rehabilitation in chronic stroke patients. Music therapy has a significant impact on improving neurological function and can change the brain’s structure and function in stroke patients (Huang et al., 2021). Thirty stroke patients received music therapy for 4 weeks. Those in the music group showed significantly better performance on the Wolf Motor Function Test, with improved timing and motion quality, compared to the mute group. Most patients in the music group could complete the task independently (Tong et al., 2015). Music can impact the brain stem, stimulate the cerebral cortex, regulate peripheral nerves, strengthen muscles, and boost physical vitality in stroke patients (Barclay et al., 2020).

## 7 Clinical trials of ischemia stroke

A randomized clinical trial showed that patients with acute ischemic stroke who received Tongxinluo along with their usual medication within 72 h of symptom onset had a better functional outcome compared to those who received a placebo (Dong et al., 2024). Acupuncture, as a complementary treatment, has shown benefits for ischemic stroke patients in randomized controlled trials (Zhang et al., 2015). A study using Wen Dan Decoction in ischemic stroke patients showed it helps improve and stabilize atherosclerotic plaques (Xu et al., 2015). Table 4 illustrates Clinical studies of ischemic stroke using traditional medicines and complementary medicines.

**TABLE 4:** Clinical studies of ischemic stroke using traditional medicines and complementary medicines.

S.no	Category	Types of medicines	Study type	Outcome measured	Ref
1	Traditional Medicines	Herbal Medicine	Randomised Clinical Trial	Oral capsules of Tongxinluo improve the Neuroprotection, anti-inflammatory, and antioxidative effects	Dong et al. (2024)
2	Complementary medicines	Acupuncture	Multicenter single-blinded, randomized controlled trial. Eight hundred sixty-two hospitalized patients	Wider use of acupuncture following a stroke could have a significant positive impact on health if the above prospective advantages are verified	Zhang et al. (2015)
3	Traditional Medicines	Traditional Chinese Medicines	Randomised Clinical Trial	The enhancement of hemorrhagic and ischemic strokes demonstrates WDD in this study. WDD-induced Qi-Energy may keep the blood in the cerebral arteries, halting the bleeding and stabilizing the atherosclerotic plaques	Xu et al. (2015)
4	Traditional Medicines	Herbal medicines	Randomised Placebo-controlled clinical study	This study offers proof that Di-Huang-yin-zi DHYZ may be in the ischemic region’s blood flow enhancement, anti-apoptotic, and anti-free radical mechanisms. This study will offer valuable insights into the potential efficacy of DHYZ as a patient adjuvant therapy	Yu et al. (2015)
5	Complementary medicines	Yoga	A Non-Inferiority Randomized Controlled Trial conducted using 36 patients	Yoga is beneficial in enhancing chronic post-stroke patients’ physical capabilities and mental health	Lenoir dit et al. (2024)
6	Complementary medicines	Yoga	A Pilot Controlled Study	Patients with chronic stroke may benefit from yoga’s ability to strengthen hand grips and lower blood pressure when combined with therapy. Age and sex may also have an impact on rehabilitation outcomes	Lai et al. (2023)
7	Traditional medicines	Ayurveda	A prospective study	Self-selected patients who were 1-month post-stroke reported that they tolerated Ayurvedic massage well. These self-selected patients had improved their standing and discharge mobility more quickly	Sankaran et al. (2019)
8	Complementary medicine	Aromatherapy	Randomised clinical trial study using 70 patients	In addition to lowering malondialdehyde and raising antioxidant levels, the use of lavender 10% essential oil helped patients with ischemic stroke symptoms such as confusion, speech difficulties, and muscle weakness	Salehi et al. (2019)
9	Traditional medicine	Traditional Chinese medicine	Randomized Clinical Trial	Xuesaitong soft capsules raised the chance of functional independence at 3 months in ischemic stroke patients in this randomized clinical trial, suggesting that this could be a safe and efficient alternative treatment to enhance prognosis in this population	Wu et al. (2023)
10	Traditional medicine	Herbal medicines	Randomized clinical trial Over the course of the 4-day investigation, the control group received standard stroke care, whereas the saffron group received standard care along with a 400 mg/day (200 mg twice day) saffron capsule	Saffron significantly reduced oxidative stress markers and improved antioxidant levels in patients with acute ischemic stroke. It also led to reduction in stroke severity after 4 days. The improvements were correlated with higher GSH and TAC levels and lower MDA levels. These findings suggest saffron extract may be a promising therapeutic option for ischemic stroke recovery	Gudarzi et al. (2022)
11	Complementary medicine	Physical exercise	A randomized controlled trial	The study findings demonstrate that, compared to standard care, a 1-year exercise intervention gradually enhanced executive functioning. The medical providers to consider incorporating exercise and physical activity into the conventional secondary stroke prevention treatment for individuals with or small stroke	Deijle et al. (2024)
12	Complementary medicines	Acupuncture	Randomized Controlled Trial (n = 162)	In the subacute stage of an ischemic stroke, acupuncture seems to be safe. The health benefits of using the medication more widely could be significant if the possible advantages seen are validated in larger research in the future	Zhang et al. (2015)

## 8 Limitations and future directions

Traditional and herbal formulations often vary in preparation methods, dosages, and phytochemical profiles, leading to inconsistent therapeutic outcomes. Most evidence supporting TCIM approaches is based on preclinical (*in vitro*/*in vivo*) models, while high-quality, large-scale randomized controlled trials (RCTs) in humans remain scarce. Many studies evaluate outcomes over brief periods, failing to assess long-term safety, efficacy, or the risk of relapse. Additionally, most clinical trials involve homogenous populations, limiting the applicability of results across diverse demographics and co-morbid conditions. Potential interactions between herbal remedies and standard stroke medications are seldom explored, raising concerns about safety. Methodological flaws, such as inadequate randomization, lack of placebo controls, and absence of blinding, further compromise the reliability of findings. Studies also vary widely in their outcome measures and definitions of key clinical endpoints like “functional recovery” and “neuroprotection,” which affects comparability and reproducibility. Furthermore, several trials do not adequately justify the choice of dose, frequency, or duration of treatment. Although some studies demonstrate beneficial effects, they often lack detailed investigations into the molecular mechanisms involved, which are essential for scientific validation and regulatory approval.

To overcome these limitations, future research should prioritize the development of standardized formulations with defined phytochemical profiles and optimized dosing protocols. Multicenter, double-blind, placebo-controlled RCTs involving larger and more diverse patient populations are needed to establish clinical efficacy. Mechanistic studies employing systems biology and omics technologies (e.g., genomics, proteomics, metabolomics) can help elucidate how TCIM therapies exert their effects. Comprehensive safety evaluations, including herb-drug interaction studies and long-term toxicity assessments, are crucial. Integrative models that combine conventional and traditional medicine could support a more personalized approach to stroke management. Finally, the use of advanced imaging techniques and validated biomarkers would enable more objective and reliable assessment of therapeutic outcomes.

## 9 Conclusion

This review underscores the potential of Traditional, Complementary, and Integrative Medicine (TCIM) in addressing the multifactorial pathophysiology of ischemic stroke. The neuroprotective, anti-inflammatory, and antioxidant properties of various herbal and alternative therapies may support improved neurological recovery and reduce the risk of stroke recurrence. Integrating TCIM into conventional stroke care requires a multidisciplinary framework that fosters collaboration among clinicians, researchers, and traditional medicine practitioners. However, to enable safe and effective implementation, further rigorous studies are needed to standardise formulations, establish therapeutic efficacy, and evaluate safety profiles. Such evidence-based integration could ultimately transform the global paradigm of ischemic stroke management.
